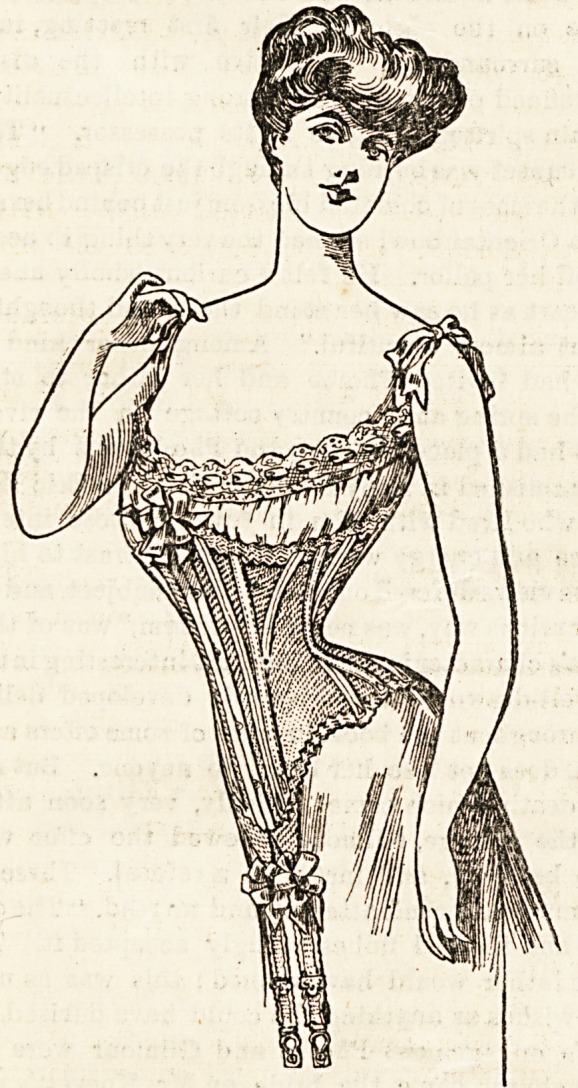# The Hospital. Nursing Section

**Published:** 1904-03-19

**Authors:** 


### The Hospital.

### Nursing Section.

Contributions for this Section of "THE HOSPITAL" should be addressed to the EDITOR, "THE HOSPITAL" NURSING SECTION, 28 & 29 Southampton Street, Strand, London, W.C

No. 912.—VOL. XXXV. *SATURDAY, MARCH* 19, 1904.

### Notes on News from the Nursing World.

### QUEEN ALEXANDRA'S IMPERIAL MILITARY NURSING SERVICE.

The Army Council have decided to grant the following increased rates of pay to members of Queen Alexandra's Imperial Military Nursing Service:—Matron-in-Chief: £300, rising by £10 annually to £350; Principal Matrons: £175, rising by £10 annually to £205; Matrons: £75, rising by £10 annually to £150; Sisters: £50, rising by £5 annually to £65; Nurses: £40, rising by £2 10s. annually to £45. These ladies also receive board and washing allowances, and matrons in charge at the larger hospitals draw extra pay, according to the magnitude of their charge.

### A BELATED HONOUR.

MRS. GEORGE KING, who was awarded the decoration of the Royal Red Cross by her late Majesty Queen Victoria for her services in the Zulu War, has just received from the Russian Government the decoration of the Russian Red Cross for her services in the Russo-Turkish War of 1877-8—an interval of 27 years. In her nursing days Mrs. King was known as Sister Janet.

### BEQUEST TO A HOSPITAL BY A NURSE.

THE announcement that the late Miss Caroline Frith, who at the time of her death was a staff nurse in St. Bartholomew's Hospital, has left to the Hospital the whole of her residuary estate of the estimated value of £2,000, is very interesting and significant. According to the will, which was recently proved, Miss Frith made the bequest in order to show her appreciation of her experiences at St. Bartholomew's, and of the kindness and consideration accorded to her during her four years' residence in the institution. The feeling of gratitude which dictated the bequest cannot be too warmly praised. Nurses do not often possess private fortunes to leave to anyone, but it would be well if the spirit that influenced Miss Frith more frequently prevailed.

### RETIREMENT OF THE MATRON OF CHORLTON UNION HOSPITAL.

WE understand that for urgent family reasons, Miss Rawson has resigned the position of Matron of the Chorlton Union Hospital, which she has held with great distinction during the past nine years. Miss Rawson was trained at Brownlow Hill Workhouse Infirmary, Liverpool, and worked at the Birmingham Union Infirmary under Miss Gibson. For the last two years of her work at Birmingham she was Superintendent of night nurses. The Chorlton Union Board of Guardians, on her appointment as Hospital Matron, inaugurated that entire reform of hospital nursing and treatment of the sick poor that has made the reputation of the institution at West Didsbury. In Miss Rawson they found a woman with a genius for organisation, a power of work, and a strong personality. No labour daunted her, and she succeeded in inspiring her staff with her own spirit and devotion to the higher ideals of the profession to which she has given the best working years of her life. In every possible way Miss Rawson has expended her time and ability to help forward the movement for improving the specific training and the professional status of nurses under the Poor Law. She will take with her into her retirement the whole-hearted affection of the nursing staff. We trust that the high standard of skilled nursing and the kind treatment of the sick poor which, under Miss Rawson, has become a tradition at the Chorlton Union Hospital will be fully maintained by her successor.

### A SWEEPING ALLEGATION.

COMPLAINTS reach us that at one of the large London Hospitals "the matron does not look after the health of her staff." The basis of the allegation appears to be that a probationer has been kept on as "a special" to a case of enteric for three months without being allowed a single day off; and it is alleged that an attack of pleurisy, from which the nurse is suffering, is "the result of overwork and want of proper care." Even assuming the accuracy of these statements, which, of course, are properly authenticated, they do not justify such a sweeping charge. To allow a probationer to continue for such a long spell of anxious work without insisting upon her taking adequate relief would show want of consideration by some one, but it cannot reasonably be expected that the matron of a large hospital should be acquainted with the exact work of each probationer, and the length of time during which she has been engaged on it.

### HOW TO KEEP ST. PATRICK'S DAY.

IN the 27th annual report of the St. Patrick's Nurses' Home at Dublin reference is made to an interesting and important new development. The St. Patrick's Nurses were instrumental some little time ago in starting a fund having in view the establishment of a small home where little convalescents could be received in order that the roses might be brought back to their faces after many weeks of suffering, the only condition necessary for admittance being that they were not more than twelve years of age, and that they had been cared for by one of the Queen's Jubilee Nurses. A subscription list was started, and the fund is growing steadily, considerably over £100 having been collected, principally in small sums, irrespective of class or creed. The time has now come, however, when, as it is pointed out in the report, work must move on larger lines or—fail. A committee of management has been appointed, and several influential ladies have promised support. Perhaps there may be Irish people in England, or English people willing to help Irish little children to enjoy a month of nursing and sunshine, instead of being allowed to fade away in Dublin dens, who would like to commemorate St. Patrick's Day this week by forwarding a contribution to Miss Berry, District Superintendent, at the St. Patrick's Nurses' Home.

### THE ACLAND HOME AND DISTRICT NURSES.

There was a long discussion at the general meeting of subscribers to the Acland Medical and Surgical Home, and the Sarah Acland Memorial District Nurses, which was held at Oxford the other day for the purpose of receiving the report of the special committee appointed to consider whether any, and if so, what change is desirable in the relations between the work of the District Nurses and that of the Acland Home, and whether any, and if so, what alterations are required in the constitution of the District Nurses and the Home, and the rules regulating the same. As the Warden of New College, who presided, said, they met to receive and, if they thought well, to approve of what was practically a new constitution of the Home. No opposition was offered to the recommendations of the special committee, and the constitution and rules were adopted. Several speeches were then made in reference to the resignation of several ladies from the committee, and Mrs. Furneaux explained why it had become essential to transfer the District Nurses to a separate Home. Miss Acland mentioned that the receipts of the Acland Home were last year £70 in excess of any previous year, and that the receipts from private nursing were £117 more, figures which she thought spoke well for the management. This is in itself an excellent reason why, in future all subscriptions should be given to the District Nursing Fund unless otherwise specified.

### NEW HOME AT YORK COUNTY HOSPITAL.

Last week the new nurses' home at York County Hospital was opened by Lord Herries. It is in the form of a new wing projecting at right angles to the main building, and at the end of the Watt Memorial wing. The home is connected with the hospital by a paved and glazed covered way which faces south to the garden, and provides a pleasant promenade for the nurses. It contains large and cheerful sitting-rooms for the sisters and nurses, and separate bedrooms for the members of the nursing staff. For the embellishment of the rooms and corridors a number of pictures and engravings have been presented, and the furnishing is on lines consistent with comfort as well as with economy. The total cost is £4,486, towards which a grant of £2,000 was made some time ago from the Marriott bequest.

### MORE NURSES WANTED AT WOLVERHAMPTON HOSPITAL.

At the annual meeting of the Board of Management of Wolverhampton General Hospital, Dr. Deanesly strongly urged the importance of making the acquisition of the Sister Dora Home an occasion for improvements which are much needed in the hospital. He pointed out that, in consequence of the increase in the number of in-patients, "an enormous amount of additional work had been thrown upon the medical and nursing staff," and contended that the most urgent want was an augmentation of the nursing staff and the provision of better accommodation for them. The approval with which his remarks were received justify the conclusion that his advice will be followed by action.

### A SLIP OF THE TONGUE.

A district nurse at Tavistock has been fined half-a-crown and costs for making a false declaration that a child of a patient, born alive in January, was still-born. The coroner's jury had previously expressed a belief that the declaration was due to a mere slip of the tongue, and though the magistrates convicted, they added that they did not think there was any evil intent. The nurse bears the highest character in the town; it was the first death she had ever registered; and the document had not been read over to her she stated, or she would, she thought, have noticed the mistake. It is impossible to be too careful in signing documents, and no one should think of appending a signature to one who has not mastered its contents.

### A BIG LOSS AT BIRMINGHAM

At the annual meeting of the Birmingham District Nursing Society it was stated that during the past year 143 cases more than in 1902 were attended, and 2,442 more visits paid by the 17 nurses. The latter also did much valuable work in visiting schools and attending to children's cuts, burns, and bruises. Unfortunately, the financial condition is not satisfactory, there being a loss of £306 upon the 12 months' working. This deficiency is larger than it would have been if the Hospital Saturday Committee had been able to continue the subscription of £100 which they formerly paid for work amongst their subscribers. We fear that the good done by the nurses is but slightly recognised by the wealthier inhabitants of the Midland City. Arrangements were made during the past year to receive at the homes of the Society probationers for the Queen Victoria Jubilee Institution for Nurses and in consideration of a premium to allow them to visit with members of the staff in order to gain experience.

### NURSES' MISSIONARY UNION.

On Tuesday last week an interesting evening was spent in connection with the Nurses' Missionary Union, at 124, Harley Street, where Mr. and Mrs. McAdam Eccles hospitably entertained a specially-invited number of nurses, representing many of the London Hospitals. The object of the gathering was to discuss the possibilities and difficulties of forming and sustaining Christian and Missionary interest among hospital nurses. Each nurse was previously supplied with an agenda of the points to be discussed, and all, therefore, came prepared to tell of the special difficulties and opportunities in their own hospital. Mrs. Howard Taylor, of China, gave a short and stirring account of Missionary work in China, in which she described the appalling ignorance and cruelty of native medical and surgical practice in that country, and showed the consequently illimitable scope of blessing and usefulness lying before a trained Christian nurse in the Far East.

### WHY THE NURSES RESIGNED AT CORK DISTRICT HOSPITAL.

In our issue of February 27, we referred to the resignation of three trained night nurses at the District Hospital, under the control of the Cork Board of Guardians, and mentioned that no explanation of their action was given. It will be seen in our correspondence columns that the nurses themselves now explain why they relinquished their posts. They state that their rations, which at first were first-class, were partly cut off; that, though having an average number of 120 patients under their individual charge every night, they were never allowed an hour off duty unless they supplied substitutes at their own expense; and that in the case of one of them, an application for increase of salary, after two and a half years' service, was not noticed. If these are the facts of the case, we think that the three nurses were not only justified in resigning, but that they were treated with great want of consideration by the Cork Guardians.

### THE DEATH OF MADAME BRON.

We regret to hear of the death of Madame Alice Bron. This devoted Belgian nurse, it will be remembered, had something to say in our columns about her experiences in nursing sick and wounded Boers and British soldiers. When she went out to South Africa her intention was to devote herself to the Boers in the Transvaal, but she subsequently felt constrained to abandon their cause and assist to minister to our men at the Cape. She became very popular amongst our sufferers, and we have no doubt that those who were nursed by her will be sorry to know that she has passed away so prematurely.

### A FREE NURSE WITH A PEDLAR'S PERMIT.

At Manchester City Police Court last week a woman of middle age, named Gertrude Bishop, who wore a nurse's costume of grey, with white bonnet, strings, and bow in the dock, was sent to prison for 14 days for obtaining contributions for a charitable organisation which has no existence. The alleged organisation was described on the outside of a pamphlet as "Mrs. John David Bishop's British-Israel, Anglo-Israel Hebrew Sisters. Free Nurses to the Poor," and it was stated by the prisoner that it was established in Manchester in July 1887 in honour of the Jubilee of the late Queen Victoria. But the evidence showed that the organisation had no existence, and that the woman Bishop had no right to call herself a nurse. In her defence she claimed to possess a license, and said that if any one asked her to nurse them she did so without charge. The license, however, turned out to be only a pedlar's permit, and the pamphlet on examination proved to be the copy of a sermon on "Britain and Prophecy," which had no connection with the supposed nursing institution. One of the witnesses deposed that she bought a pamphlet from the prisoner, who said, "We are the only nurses who are not collected for. We nurse the poor free of charge, and we are all domesticated."

### MIDWIFERY TRAINING AND CROYDON INFIRMARY.

The Croydon Guardians were recently asked by the Association for Promoting the Training and Supply of Midwives whether they could not offer any facilities for the training of midwives. At the instance of the Medical Superintendent, the Guardians have replied that as there is only sufficient accommodation at the Union Infirmary for training the Board's own probationers, it is impossible to comply with the request. We fear that this will often prove to be the case.

### EXCELLENT PROGRESS AT HASTINGS.

In contrast to the financial position of so many nursing organisations, that of the Hastings District Nursing Association is highly satisfactory. The annual report, of which Lord Brassey moved the adoption at the annual meeting, shows that between 9,000 and 10,000 visits were paid by the nurses in 1903—an increasing number—and that the balance of income over expenditure was £85 as against £62. This is a healthy condition of things, which attests the energy and good management of the committee as well as the devotion of the nurses. But the point we desire to emphasise is that a result which is achieved at Hastings—good nursing and financial solvency—can be obtained in other towns of similar size and with similar sources to be tapped.

### SHORT ITEMS.

As the result of allegations of neglect of patients at the Slough Workhouse Infirmary, the Eton Board of Guardians have appointed a committee to consider the desirability of increasing the staff of nurses.—On Thursday evening last week a successful entertainment, organised by Miss Lloyd-Jones, was given to the in-patients of the Cancer Hospital, Fulham Road.—Miss Jessie Torrance has resigned her appointment as Matron of the Memorial Cottage Hospital, St. Andrew's, which she has held for the past six years.

### The Nursing Outlook.

"From magnanimity, all fear above;
From nobler recompense, above applause,
Which owes to man's short outlook all its charm."

### NURSING IN GREAT BRITAIN.

### II.—THE NURSE AND THE PUBLIC.

NOTWITHSTANDING the revolution which has taken place in nursing during the last 20 years, the trained nurse of to-day is growing less, rather than more, popular with the public. This decrease in popularity is mainly due to the fact that the nurse-training schools have always refused to unite to secure an uniform standard of education, an identical basis of examination, and a single certificate, the possession of which would guarantee the public that the nurse was, in a technical sense at any rate, fully qualified in all branches, and might so be safely trusted to take the full nursing care of a patient.

The second cause of dissatisfaction arises from the circumstance that when once a nurse has obtained a certificate of training from somewhere, however imperfect and incomplete that training may be, she can go into the market and readily obtain employment under the patronage of private adventure homes which supply nurses, or through other agencies. Thus the nursing body comprises at the present moment a very large number of women who could not be expected to pass the tests which any central examining board must impose as the minimum standard for a trained nurse.

Amongst the better type of the smaller institutions, where the authorities may conscientiously endeavour to do their best for the probationers they receive, with or without payment, it is impossible for them with the limited means at their disposal, to afford any adequate training, in the proper meaning of that word. The young women who apply possess little or no knowledge of the requirements of a nurse or they would not agree to enter one of these institutions when desirous to qualify as a trained nurse. The number of the smaller hospitals and institutions is so great, however, that they do in fact turn out every year a large number of women, who, in virtue of the imperfect experience they have gained, call themselves nurses, and have little difficulty in finding employment. The consequence is great detriment to the public, to the properly-trained nurse, and to the position of nursing in this country to-day.

Nor does the evil lie entirely at the door of the smaller institutions which receive nurses for training, for amongst the larger hospitals the standard of training varies so greatly, that a nurse may give three years of her life, and obtain her certificate from one of these great hospitals, without having received any real training or experience in several special branches of her profession. In other words, such a nurse may see but very little beyond medical and surgical nursing, owing to the fact that the training of each individual nurse, under the present circumstances, may depend entirely upon the will of the individual matron in charge of the hospital with which she becomes associated. This clearly points to a want of public spirit on the part of those who are responsible for the continuance of this state of things. Over and over again a few of the more public-spirited and best-informed hospital authorities have approached the representatives of the greater hospitals with a view to secure the reforms in nursing which are urgently called for. These efforts have invariably failed because the majority of the larger institutions, or those responsible for their management, have refrained from supporting a scheme to raise the standard of education and training for nurses all over the country. They hold to the view that so far as the system in force at their particular institution is concerned, everything is of the best, and some of them undoubtedly make a very considerable sum each year from the earnings of their private nursing staff. In such circumstances the wiser managers regret that the majority are not prepared to take the first practical step in the direction of a general scheme which would raise the standard of nursing and protect the public from the present crying evils which attach to nursing in Great Britain to-day.

There is one other reason why nurses are becoming less popular, and it arises out of those already given. In the present absence of any standard of training, the public find in practice that some nurses, supplied to private families, so comport themselves that it is a worry to retain them in the house. This experience has made the public begin to realise that, granted there was an uniform standard of training, a central board of examination, and one standard certificate which they could accept as evidence of the technical efficiency of the holder, they would still require some guarantee of the character of the nurse before they could receive her into the family with perfect confidence. This difficulty is the rock upon which the nurse-training schools have split in the past. They recognise that in a nurse who has to be constantly associated with the patient and the family, character and womanliness are the two essential qualities, which no certificate of training can guarantee. Hence those who know most about nurses and nursing, though they may have originally opposed registration because it is not a panacea for the existing evils, are coming to see that if the nurse-training schools will not combine to provide uniformity in the matters referred to, registration must be accepted as the primary step, for it can at any rate secure that every registered nurse shall possess the minimum standard of technical qualifications necessary for the discharge of her duties in a public institution or private household.

### The Modern Nursing of Consumption.

By Dr. JANE H. WALKER, Physician to the New Hospital for Women, London, and Medical Superintendant of the East Anglian Sanatorium, Nayland, Suffolk.

### LECTURE I.

MODERN theories as to the causation of disease in many instances involve modifications in treatment, and treatment includes nursing as an essential point. In no case is this more fully seen than in phthisis pulmonalis, commonly called consumption. The changes which have taken place of late years, not only in our views as to the causation of the disease, but also in our treatment, have followed upon the discovery of the bacterial origin of tuberculosis. Tuberculosis, wherever it may appear in the body, is due to the inroads of the tubercle bacillus. When, from some cause, perhaps an initial injury, a general lowering of the vitality, or it may be an inherited predisposition, the tubercle bacilli find an entrance to the body and a suitable soil, they settle down, and by irritation of the surrounding tissues lead to the formation of hard nodules or tubercles. Around these nodules we find an area of inflammation and congestion, and f the nodules be situated in the external part of the lung, this congested spot may border so nearly on the pleura (or covering of the lung) that localised pleurisy may follow, giving rise to the pain which is a frequent early sign of phthisis.

These nodules may remain simply as hard tubercles, and the mischief go no further. Very many healthy people who are unaware that they have ever had anything the matter with their lungs, have at one time or another had small areas of lung affected by tuberculosis, and it has been estimated by some observers from the results of post-mortem examinations, that from 20 to 30 per cent. of patients dying from quite other causes have old healed tubercles in their lungs.

On the other hand, owing partly to the poisons (toxins) manufactured by the bacilli, partly to the death of the cells caused by the blood supply to the nodules being cut off, the centres of these tubercles may break down, soft pus-like (caseous) material being formed, which process gradually going on, leads to the formation of definite small cavities. These cavities, with their contained caseous material, may run together forming larger and larger cavities, or may, and generally do, rupture into the air-cells of the lungs, giving rise to much expectoration, the patient constantly bringing up masses of this caseous matter, mixed with particles of broken-down lung substance, and the secretion of the irritated bronchi.

Besides the tubercle bacilli, other organisms may find entrance from the air; the most usual of these are the ordinary strepto- and staphylococci of pus formation. The exact part which these organisms play in the process of consumption is still a matter of discussion, but we are justified, I think, at present in saying that, where high temperature, night sweats, rapid pulse and emaciation are marked features of the illness, there these organisms will generally be found microscopically. Indeed it is thought by many that the diminished temperature, the cessation of night sweats, and gain in weight which are such striking features of the open-air treatment of phthisis, are really due to the diminution in virulence or total disappearance of these organisms under the influence of fresh air. A case of consumption may progress towards death either (1) by more and more of the lung being excavated by tubercles, and more and more of these tubercles breaking down to form cavities, until so little lung is left that the patient dies from mixed dyspnœa and general weakness, or (2) the patient may die simply from asthenia, the pulse and temperature getting worse, the general weakness progressing, death resulting not so much from the local lesion as from the effects of the bacillary toxins; or (3) one or more large blood-vessels may run through a cavity, and the surrounding lung being eaten away, the unsupported vessels give way, or the vessel walls may be ulcerated through by the tubercular lesion; in either case a sudden and fatal hæmorrhage may occur. The violent later hæmorrhages of phthisis are generally due to the bursting of a vessel running through a cavity.

Recovery may ensue by the patient's resisting powers being sufficient to enable him to overcome the toxins secreted, and instead of cavitation advancing progressively, the contents of the cavity are got rid of by expectoration, the walls cicatrise, the tubercles become fibrous, and we have a progressive cure. The portion of lung which was excavated is of no further use for breathing purposes, the walls of the cavity fall in, and we have, in a healed cavity, a large fibrous scar, which weakens the patient's breathing powers. Though, owing to the fact that we are provided with a considerably larger amount of lung tissue than is necessary for ordinary breathing purposes, it is only when the portion of lung destroyed is very large, or when it is much bound down to the chest walls by pleural adhesions, that the respiratory powers are seriously hampered by the presence of cicatrised cavities.

In an old fibrous and apparently-healed nodule a few tubercle bacilli may remain enclosed, capable, if the patient be run down in health, or has influenza, typhoid, or any other debilitating disease, of again becoming active. Therefore, any patient who has ever had phthisis should be careful to keep himself, so far as possible, up to the mark, and should take especial care of his health if he be subjected to any abnormal strain in the way of illness, or very hard mental or physical work.

If tubercle bacilli be present in the sputum of a patient, that patient forms a source of infection when the sputum is dry; for the tubercle bacilli can be carried about when dry in the air, and therefore the air of the patient's room, the dust of the room, the patient's bedding and clothing are all liable to be infected. This is an important fact to remember in the nursing of consumptives. The tubercle bacilli are not very resistant to light and are said to be rapidly destroyed by direct sunlight, hence the importance of light rooms, and plenty of sunlight and fresh air, and of providing suitable receptacles for all sputum and also the great importance of keeping the patient's person, bedding, and room clean.

The occupation followed by a patient is of importance, owing to this infectivity of dried sputum. Ill-ventilated rooms and crowded workshops and dusty occupations have a threefold danger. In the first place the tubercle bacilli live long in a dark dusty atmosphere; in the second the worker's vitality suffers from bad air; in the third the lungs are liable to be irritated and inflamed by dust, and so give an easy entrance to the bacilli.

In crowded workshops we have not only all these dangers, but, where there are a considerable number of persons (especially persons of the lower classes) together, there are sure to be some sufferers from phthisis, and with the disgusting habit of spitting at present prevalent, the floors are certain to be infected to some extent with the bacilli.

At the risk of boring our readers by reiterating an oft-told tale, we must at this point draw attention to Dr. Henry MacCormac's paper, published so long ago as 1855. His treatise drew attention to the absolutely poisonous and infective character of re-breathed air. He pointed out the prevalence of consumption where the conditions of a vitiated atmosphere exist, and the improvement in the state of a consumptive individual when he is removed from bad air and surroundings and also to the possibility of preventing consumption by an abundant supply of pure fresh air. His paper, which everyone now would consider common knowledge, was then treated with scorn by his colleagues, and when he read it his hearers refused to pass a vote of thanks to him.

His contention, stated in other words, is that the great cause of consumption is overcrowding, and that wherever the conditions of overcrowding exist there is consumption rampant. This is the case wherever the condition obtains, whether in the Sahara Desert or the blackest slums of Whitechapel.

We have above drawn attention to the evil results consequent on promiscuous spitting. As bearing further on this point we notice [1] that Doctor Dieudonné, of Würzburg, has recently written a paper on the etiology of tuberculosis in childhood. His remarks are based on some observations which show that tuberculosis is most common in children who are between one and two years old, but forms on the whole a rare occurrence in the first year of life, and that in those who are between three and four years old the percentage of cases of tuberculosis is decidedly lower.

Dr. Dieudonné examined the dirt on the hands and the secretions of the nose of a number of children whose ages varied from nine months to three and a half years. He almost invariably found bacilli which were bacteriologically proved to be tubercle bacilli. From these observations he concludes that the great frequency of tuberculosis in children is largely due to the fact that young children crawl on the floors on their hands and knees. A child soils his fingers with dirt containing tubercle bacilli, and then contaminates his nose and mouth. During the first year of life a child is carried about in his mother's arms, and after two, or two and a half, he has generally got the use of his legs, and runs instead of crawling, which is confirmatory of the conclusions drawn from the fact that tuberculosis is commonest during the second year of life. It may further be pointed out that the form of tuberculosis most prevalent in the young is the abdominal, which would result from the introduction of the bacilli directly into the alimentary tract. A fertile method of infection we believe to exist in the rubber "comforter" which children of the poor, in the intervals of dropping on the floor, so constantly suck. These "comforters" of course tend to become infected with the organisms, if there be any sputum on the carpets or boards. It is perhaps premature to draw positive conclusions from Doctor Dieudonné's observations, but they serve to impress upon us the moral, which is not: carry babies about till they are three years old; but that the evils of indiscriminate spitting may be more far reaching than we have ever anticipated.

Though phthisis is undoubtedly a disease spread by infection from the sufferers, still we are all at the present time agreed that the disease is infectious to a limited extent only. If ordinary precautions be taken there is no risk to those in attendance on phthisis patients. A reduced state of health, a prolonged exposure to the disease in unhygienic surroundings, such as we find in the stuffy overheated rooms of rich and poor alike, but especially of the poor, are common antecedents to an attack. Both nurses and doctors in consumption hospitals must be constantly exposed to the infection, yet we rarely hear of any of the staff contracting the disease. It is indeed to be questioned whether the great stress laid on the infectiousness of consumption in both lay and medical writings has not done as much harm as good. It is a fact that, whereas formerly the consumptive was regarded as no more infectious than if he had a wooden leg, he is now by many thought to be as infectious as if he had scarlet fever or measles. The truth lies between these two views, provided certain simple measures are carefully carried out, which measures we shall deal with later. There is no danger to those in charge of a case of phthisis.

Some persons lay stress upon the importance of nurses for phthisis cases being specially physically strong. On the other hand it is sometimes considered an advantage for the doctor in a sanatorium to have been himself a patient, and to have gone through the necessary routine and discipline. There is a good deal to be said for this idea, and whatever it be worth it holds just as much for the nurses. We have ourselves now many nurses employed who have been former patients, and there is no doubt that they are peculiarly well fitted to realise the importance of the details of nursing consumption cases. Certainly many anæmic and otherwise "crocky" women improve marvellously while nursing in sanatoria; the fresh air, the abundant food, the regular hours, all combine to keep the nurses at a physically high level of health, and there is equally certainly no need for them to be especially robust.

[1] Brit. Med. Jour., Sept. 26, 1903 (p. 750).

### State Registration of Nurses.

The following manifesto is in course of circulation. It will be noticed that only eleven signatures are attached, of which six represent members of the Central Hospital Council for London, to which 35 members were appointed as representatives of the 12 great Nurse Training Schools in London. It will further be observed that the signatories only include three matrons and Lady Roberts.

We, the undersigned, feel called upon to make the following statement in view of the steps that are being taken to obtain, by Act of Parliament, a State Registration of Nurses.

The objects claimed for such registration are: (*a*) The benefit to the public, who would, it is said, be enabled to ascertain from the register the competency of the nurse employed, and would be protected from unskilled and incompetent persons. (*b*) The benefit to the "trained nurses" themselves, who are alleged to be placed at a disadvantage by the employment of imperfectly trained persons.

We believe that these claims are mistaken, and that any system of State registration would be detrimental to the public and harmful to the nurses themselves, for the following reasons:—

1. Inasmuch as any system of registration must be based on the results of testing by examination the technical capabilities of a nurse, it of necessity raises to a predominant position this side of her work, and leaves entirely unconsidered those personal qualities upon which her main value depends, such as good temper, manner, tact, discretion, patience, and unselfish womanliness. It is these characteristics which cannot be ascertained by examination, and which no system of registration can include.

The experience of those concerned in the training of nurses and supplying them to the public shows that it is the want of these qualities in a nurse which give rise to

### STATE REGISTRATION OF NURSES—*Continued.*

complaints on the part of patients and their friends. It is seldom that a want of adequate technical training is the ground of fault-finding.

Moreover, it is the difference in the comparative value of the technical skill and the personal qualities in the making of a nurse which constitutes the essential difference between her and a doctor as regards the applicability of a system of registration, and renders the analogy, so often made, entirely fallacious. A doctor's technical knowledge takes many years to acquire, and his education is tested at various stages by authorised bodies, and, however important his personal character may be, it is for his skill and knowledge primarily that he is consulted. But, without desiring to underrate the importance of the technical knowledge of the nurse, it is certain that—apart from a speciality such as midwifery—the extent of this knowledge is secondary in importance to her personal character.

It is well known to many of the signatories that not a few women who have done extremely well in examinations, have quite failed to make good nurses, or such as could with confidence be sent into private families.

No one would engage a governess, or even a domestic servant, simply because her name is on a register without inquiring into her character as distinct from her ability to perform her specific duties. *A fortiori*, the same inquiry should be made before engaging a nurse. No register would in either case furnish the requisite information.

2. A State register of nurses, far from being a security to the public, would be an actual source of danger, since an utterly unsuitable woman, simply because she has passed an examination, would be entitled to be on the register, which it is claimed would certify to the nurse's fitness.

3. Great difficulty, personal odium, and possibly the expense of defending an action for libel, would attend anyone seeking to have a nurse's name removed from the register even if she were notoriously bad. Shortcomings sufficient to disqualify her as a nurse would be almost sure to be passed over, and a really bad nurse might, and many would, be going about "hall-marked" as fit to be employed. The public would be lulled into a false sense of security, being led to believe that the register would protect them from incompetent and undesirable nurses.

4. In our opinion it is not advisable that there should be an uniform training made compulsory on all nurses such as a State registration would require. To supply the manifold needs of patients and to meet the very different conditions under which nursing of all sorts and kinds has to be done, a variety of nursing knowledge and experience is requisite, and a large number of women, trained only in certain directions and who would not comply with the conditions imposed by registration, satisfactorily supply what is wanted. To exclude such from following their occupation, as a State register more or less aims at, would be as unjust as it would be impracticable.

5. If nurses are to be registered on their technical qualifications (and it is conceded even by the advocates of registration that nothing else can be "registered") it is inevitable that they will concentrate their efforts on the attainment of the technical knowledge which is thus made the first essential. From the beginning of their training they will deem the passing of examinations to be of primary importance. Those who realise that the ultimate success of a nurse must depend upon her personal suitability for her work, already deprecate the growing tendency to attach undue importance to the passing of examinations at the expense of the cultivation of those qualities of power of observation, of sympathy, cheerfulness, and self-control without which the services of a technically trained nurse can never be acceptable to a patient.

6. A State register such as is proposed would tend to lower the status of the best nurses, partly from their association thereon with those persons who, from defects of character or performance, ought to be removed from the register, but have not been so for the reasons stated. Further, if by the imposition of an unduly high standard of examination, the best nurses (*i.e.*, those able to pass such examination) may be said to be protected, this would be attained by the exclusion from the nurses' calling of a large number of women who could perfectly well fill many situations for which their services were suitable. If, on the other hand, an unduly low standard be set, the women most competent at examinations would be placed on the same level as the less capable, and those best qualified would lose most.

When in 1893 a scheme for the registration of nurses was promoted, a similar protest to this was issued, signed by Miss Florence Nightingale, and representatives of almost all the large London nurse-training schools as well as most of those in the Provinces, and we know that to-day Miss Nightingale's opinion remains the same as it then was, that as the personal qualities, which are of first importance in a nurse, cannot be registered, it would be misleading to allow nurses or the public to imagine that any scheme of State registration would indicate the fitness of any woman registered to act as a desirable attendant on the sick.

It should not be forgotten that all important hospitals give to the nurses trained in their wards, and whose work has been well done, certificates of service after the ordinary term of three or four years has elapsed. These certificates are sufficient testimony of technical knowledge and experience, and would not be improved upon by a registration, or examination, by persons who had no experience of the actual conduct of the nurse during her period of service.

Sydney Holland, Chairman of the London, Poplar and Tilbury Hospitals, and Member of Queen Alexandra's Imperial Military Nursing Service Board, and of the Council of the Queen's Jubilee Institute for Nurses.

Charlus Burt, Chairman of the Royal Free Hospital and of the Central Hospital Council for London.

Nora Roberts, Vice-President of Queen Alexandra's Imperial Military Nursing Service Board.

J. G. Wainwright, Treasurer of St. Thomas's Hospital.

Henry Bonham-Carter, Chairman of the Nightingale Nursing School.

W. H. Allchin, M.D., F.R.C.P., Senior Physician to Westminster Hospital and to the Westminster Training School and Home for Nurses.

Norman Moore, M.D., F.R.C.P., Physician to St. Bartholomew's Hospital.

Eva C. E. Lückes, Matron of the London Hospital.

Katherine Monk, Sister Matron of King's College Hospital and Member of Queen Alexandra's Imperial Military Nursing Service Board.

Mabel H. Cave, Matron of Westminster Hospital and Member of Queen Alexandra's Imperial Military Nursing Service Board.

Herbert Larder, Medical Superintendent, Whitechapel Infirmary.

March 1904.

### Danger to Life from Burns and Scalds.

### EXAMINATION QUESTIONS FOR NURSES.

The question was as follows: Define the different degrees of danger to life, from various kinds of severe burns and scalds, and the means you would employ to obviate fatal results.

### First Prize.

The degree of danger to life resulting from severe burns and scalds varies to a great extent with each individual case. In accidents of this class the secondary cause of death may be "shock," inflammation of lungs or kidneys, exhaustion from the very copious discharge or septic absorption.

Shock is most fatal to young children, the aged, and those of low vitality. The patient must be wrapped in hot blankets. Hot-water bottles must be placed near him in the bed, a stimulant given, and great quiet maintained.

Inflammation of the lungs and kidneys is most likely to occur where the injuries are extensive rather than deep, and on the head or trunk. The large surface of skin unable to perform its function throws additional work upon these organs, and in cases where they are already diseased the likelihood of a favourable termination is very much diminished. Should the patient survive the first stage of shock a keen watch for symptoms of inflammation must be kept, and these promptly reported. The room must be kept at an even temperature, and the patient protected from draughts. He should be assisted to lie in the most comfortable position, allowing full play to his lungs, and not entirely on his back, lying low on his pillow.

Exhaustion should be combated by light, nourishing diet and such stimulant as is ordered. All fatigue must be minimised to the utmost, and throughout the wounds must be treated with strict regard to aseptic and antiseptic principles.

A very fatal injury may result from an attempt to drink from the spout of a kettle, a not uncommon accident in the homes of the poor. Such a case needs unceasing watchfulness, as œdema of the throat may arise quite suddenly, and the child suffocate in a few minutes. Tracheotomy instruments should be in readiness, and the alarm given promptly. Nourishment must take the form of enemata while swallowing is difficult or impossible.

Paralysed patients burnt by the application of hot-water bottles may die from exhaustion due to prolonged suppuration, as burns of this nature heal slowly or not at all.

"Damaris."

### Second Prize.

In all cases of severe burns or scalds the greatest danger is from shock to the nervous system. The first thing to do is to exclude the air as soon as possible and cover the patient well round with blankets or any warm covering procurable, and place hot bottles or bricks round, and, if able to swallow, give warm coffee or soup; it is better not to give spirits, because an inflammatory fever often follows after shock; congestion of the internal organs also often follows if the patient rallies from the shock.

The shock lasts from two or three hours to 48, and fever from a week to a fortnight, which leaves the patient in a state of great exhaustion which may last for a month or more.

The most dangerous parts of the body to be affected by burns or scalds are the face, neck, chest, or abdomen; a merely superficial burn or scald extending over a large part of the body is more fatal than a limited one on the arms or legs; even if it goes nearly down to the bone, great care must be exercised on removing the burnt clothing or any foreign body from the wound.

Another danger most common with children is drinking boiling fluid out of kettles or teapots; the air passage may be scalded to such an extent as to swell up and cause death from suffocation; a medical man must be instantly summoned, as tracheotomy will in all probability have to be performed. The same precaution must be taken to prevent shock, and very cold water given in sips, or, if ice is procurable, small pieces given to suck. When a burn is produced by chemicals, strong acids, etc., whatever is done must be done instantly, as they burn through clothing and flesh rapidly; if there is plenty of water handy, plunge the part in and move it about rapidly; do not wait to remove clothing; that may be cut off while the affected part is under water; when all the corrosive substance is removed it will be treated as an ordinary burn.

"May."

### The Successful Papers.

"Damaris," an old prize-winner, succeeds in gaining the first, though it would have been better if she had mentioned the important fact that a large superficial burn is more dangerous than a smaller deep one. She evidently understands this, as shown from her remarks relative to the threatening of inflammation of the lungs and kidneys; but it would have been better to state it explicitly. Her remarks concerning the position of the patient in bed are worthy of attention. "May" wins the second prize. She mentions the different degrees of danger to be apprehended from superficial and extended and deep and restricted burns, but what she says concerning the giving of stimulants is not quite clear. In the state of shock, and often of collapse, that a badly burnt person is in, almost invariably it is necessary to give a stimulant, and there is no fear (under the circumstances) that an isolated dose of alcohol would cause inflammatory symptoms. Always try to discriminate between a broad principle and individual cases. The sentence about immersing the injury caused by chemicals is also a little vague, though her actual meaning is clear. She should have mentioned that the water must be warm, and that it is employed to float off clothing from the wound where it might be embedded, and not express herself so that it might be understood to mean a relief to pain.

### Honourable Mention.

This is gained by "Rose," "Aubrey," "Anxious," and "Brum."

"Rose's" paper is excellent, but so carelessly and badly expressed as to be unfit for printing.

### Defective Papers.

"Lanoitan" sends a good paper, but the measures he recommends for the alleviation of threatened suffocation are somewhat drastic for any but a surgeon to employ.

Three answers were sent in which have been written almost word for word from a handbook.

### Question for March.

In nursing a patient who had been operated on for stone in the bladder by the supra-pubic method, how would you arrange his bed and the dressings to insure the greatest possible degree of comfort—suppose the case to be in a household where neither money nor appliances are plentiful.

The Examiner.

---

### Rules.

The competition is open to all. Answers must not exceed 500 words, and must be written on one side of the paper only. The pseudonym, as well as the proper name and address must be written on the *same* paper, and *not on a separate sheet*. Papers may be sent in for fifteen days only from the day of the publication of the question. All illustrations strictly prohibited. Failure to comply with these rules will disqualify the candidate for competition. Prizes will be awarded for the two best answers. Papers to be sent to "The Editor," with "Examination" written on the left-hand corner of the envelope.

N.B.—The decision of the examiner is final, and no correspondence on the subject can be entertained.

In addition to two prizes honourable mention cards will be awarded to those who have sent in exceptionally good papers.

### The National Training School for District Midwives.

### MEETING AT THE MANSION HOUSE.

By invitation of the Lady Mayoress a meeting was held at the Mansion House on Monday afternoon on behalf of the proposed National Training School for District Midwives.

The Chair was taken by the Countess of Stamford, who was supported by the Bishop of London, Dr. Cullingworth, Dr. Annie McCall, and Miss Alice Gregory, to whom the initiation of the scheme is due. There was a large attendance of ladies, including the Lady Mayoress, Miss Cock, M.D., and Miss Peter, superintendent of the Queen Victoria Jubilee Institute for Nurses. Lady Stamford, remarking that the scheme on behalf of which she wished to invoke interest and sympathy was one which it was hoped would not only help to mitigate suffering and preserve life, but would also ensure the health of future generations, called upon the Bishop of London to introduce the subject.

### An Appeal to Rich Women.

The Bishop said that England was lamentably behind the chief continental countries in the matter of the training of midwives, and the Bill providing for the registration of midwives in this country having now become law, a great responsibility was laid upon the public. In his opinion a training school for midwives should be a national one, and it should receive a grant from the State. It was hardly necessary, he added, for him to say that no sort of opposition was intended to any existing agency, and he concluded by appealing to the rich women whom he saw before him, who could afford to have every comfort in illness, to enable a scheme to be set on foot which would prove that England did not intend to be left behind the other countries of Europe in this matter.

Miss Annie McCall said that the object of the meeting was to raise funds to start the proposed school. There was no doubt whatever about the need for it. From her own experience of 20 years she could safely say that not more than five per cent. of the women prepared by her for the examinations of the London Obstetrical Society devoted themselves to the practice of midwifery. This was plain proof that the profession needed to be made more attractive, and she believed that the proposed school would have that effect. The ideal was a high one, but it might be realised if the necessary funds were forthcoming.

### The Experience of Miss Gregory.

Miss Alice Gregory, who was warmly received, related her experiences as a district midwife in Somersetshire during eight years. The great evil was drunkenness; the midwife not only drank herself, but plied the patient with spirits, and in many cases the confinements were neither more nor less than drunken orgies. This constituted an evil that could not be over estimated. She had been repeatedly told that she would not be able to command even a low fee for her services, but the contrary had been proved, labouring men with small wages being quite willing to pay the eight shillings she asked, while ten shillings was not considered too much in many cases. She had had only one bad debt, a matter upon which she certainly congratulated herself. In towns, she believed, a higher fee still would be readily obtained, for people were beginning to recognise the importance of skilled attention at confinements. Provided the right centre of work were chosen, a trained woman with a small capital to start with would be quite able to maintain herself; and many ladies' committees throughout the country, in districts badly needing a midwife, were willing to pay a somewhat similar salary to that received by the Queen's nurses. This was a fact which, she hoped, might clear some difficulties from the path of those otherwise willing to enter for training at such a school as she proposed.

### The Scheme.

Miss Gregory then explained her scheme for founding a small general hospital with a maternity annexe, in some neighbourhood where it was required, the hospital to be recognised as a national training school for district midwives, where educated women should receive training in general and monthly nursing, prior to a six months' course in midwifery, the latter to include both hospital and district work. Supplementary instruction in simple hygiene, sanitation, and physiology, cottage cookery, infant feeding, the prevention of infantile diseases, and the use of such drugs and antiseptics as are permitted in midwifery should be given. The minimum course of two years should be succeeded by re-examination and instruction in modern obstetrics every three years, the central organisation supervising the midwives at their work in the intervals. The three years' course in a general hospital, followed by specialisation in maternity training, was, Miss Gregory said, frequently out of the question on account of the time and money involved; while an educated woman recoiled from facing the responsibilities of work as a midwife after the three months' course accepted as sufficient by public opinion. Moreover, it often fell to the midwife's lot to nurse the case afterwards, under medical supervision, and for this some general hospital training was essential. It might, however, be found impracticable to insist on more than a three months' specialising course, and in that case there should be a supplementary post-graduate course and examination, when a visit to the school would revive the enthusiasm, and relieve the sense of isolation consequent on long-continued work in a far-away district. It was proposed to make a beginning, probably in the south-eastern part of London—Woolwich or Deptford—by founding a small maternity home, to which no one would be admitted who had not had at least a year's general training in some recognised hospital, where the handling of the patient, the use of antiseptics, and other elementary knowledge had been gained, since these things could not be compressed into a course of a few months. It had been said that such a school would take away the work of the Central Midwives' Board, but, since it was not within the scope of that body to train or supply midwives, the objection fell to the ground. The crux of the whole question was one of expense, but if Italy, ground to the earth by taxation, insisted on a two years' training for its midwives, surely, although the subject bristled with difficulties, England ought to bestir herself in the interests of the lives of mothers and the health of the children.

Dr. Cullingworth endorsed all that Miss Gregory had said, adding, as a member of the Midwives Board, that that body had its hands quite full enough without attempting to solve the question of training and supply.

At the close of the meeting a collection was made for the inauguration of the proposed school.

### Death in Our Ranks.

The death of Nurse Priestley, of the Mansfield and Mansfield-Woodhouse District Nursing Association, occurred on Saturday, March 5, from pneumonia. For 15 years Miss Priestley worked among the poor, and her kind services were much appreciated.

### East London Nursing Society.

The annual meeting of the East London Nursing Society was held by permission of the Right Honourable the Lord Mayor, who was in the chair, at the Mansion House on Tuesday. A large number of persons were present, including the Lady Mayoress, Lady Quayle Jones, and the matrons and nurses of the Society.

After a few opening remarks as to the value of good nursing in the homes of the poor, the Lord Mayor mentioned the fact that unless additional funds were forthcoming it was feared that nurses might have to be withdrawn from one or two districts.

The Archdeacon of London, in moving the adoption of the annual report and balance-sheet, and the re-election of the Council, referred to the statistics of the Society.

Sir William Quayle Jones seconded the motion, and it was carried.

A resolution acknowledging the value of the work done by the Society and undertaking to make its claims more generally known was also proposed and adopted. During the proceedings it was mentioned that on an average each patient costs 11s. annually, and each visit—which averages 12 minutes—costs 6d. It was hoped that the sum of 11s. might be reduced to 10s., so that each subscriber of 10s. would be able to realise that he had been responsible for the relief of one sick person for a whole year.

Dr. Robert Hutchison, who also supported the motion, in giving his experience of the work, said that the Society's nurses were an extension of the work of the London Hospital. The hospital was able to discharge patients earlier than they otherwise would do if they did not know of the after-care that would be bestowed upon them by the nurses. This also applied to the out-patient department of the hospital, which was greatly helped by the Society's work. He considered that the district nurse was the intelligence officer of the doctor. He looked forward to the day when doctors would be paid for keeping people well, not only for healing the sick. District nurses, he thought, were not merely ministers of healing; they were officers of health. The people were ready to listen to the laws of health by the examples of the apostles of health themselves; and nurses spread abroad these rules of health.

The Rev. H. Maynard, Vicar of St. Peter's, Bethnal Green, who also supported the motion, said that the nurses were a lever by which the clergyman was often able to get to the hearts of the people. Such influence frequently resulted in a change of habit and life. Nurses' sympathy and skill were not generally wasted, and he only remembered two ungrateful cases. In one the people were so interested in the nurse and her instruments that they made off with a bag of her implements, which she had deposited in the house, leaving the patient behind them! The other "case" indignantly requested that since the nurse gave no coals or other relief, she was not wanted. These were the only exceptions.

Towards the close of the meeting attention was called to the fact that the East London Nursing Society, which was established in 1868, was the mother of all the district nursing societies in London. It was affiliated to Queen Victoria's Jubilee Institute for Nurses in January 1891.

The Chairman of the Society, Mr. John Tennant, regretted that no Nonconformist ministers were able to be present at the meeting. He added that last year the Chief Rabbi proved his interest in the Society by being present on the occasion.

### Lord Lytton on District Nursing.

The fourteenth annual meeting of the Hammersmith and Fulham District Nursing Association was held on Tuesday evening in Fulham Town Hall, under the chairmanship of Mr. J. A. Curtis, Mayor.

The adoption of the annual report was moved by Mrs. Creighton, who drew attention to the fact that 24,000 visits had been paid by the nurses in the past year, making an average of about 1,500 visits of each member of the staff. More nurses were wanted to cope with the needs of the district, and this involved increased financial support. The system of collecting boxes and books was an excellent one, since by its means opportunity was afforded to the poor to contribute. £30 had been collected in this way by Mrs. Levy, whose example was worthy of emulation. Referring to the work in the Board Schools, Mrs. Creighton said that it was one of the most useful "phases of district nursing." Co-operation with other local charities was an admirable feature of the Association, patients frequently being assisted after recovery from illness by its means.

The motion was seconded by the Rev. H. Vincent, Rural Dean of Fulham, who alluded to the Association as one of the best friends of the clergy. The educational value of the nurses' work could hardly be over estimated. The Association worked over a very large area, and possibly as its scope increased it might be found advisable to have a branch Home in the Wandsworth Bridge Road district.

### Speech by Lord Lytton.

Lord Lytton proposed the re-election of the council. The coming of the nurse, he said, worked wonders. In a country village with which he was associated the decision to subscribe for a district nurse brought about a friendly feeling among the inhabitants, while the homes of the poor were transformed by her visits. If this were so in a small parish, the effect should be greater still in a large district. He thought it argued an extraordinary amount of indifference to the needs of the locality that an Association like this, after 14 years' work, received such inadequate support. That was a matter that should be remedied. In a country like England, where civilisation had reached a high stage of development, there was a danger lest the common blessings of life should be accepted as mere matter of course, for which no gratitude was necessary. That danger had to be faced and overcome if really good work was to be done. Appeals for help came to most people in so many diffierent forms that it was often difficult to choose which were most deserving. A friend of his to whom he appealed for help for a charitable cause said he felt like a visior to the parrot house at the Zoological Gardens, so deafened by the noise and distracted by the varied attractions of the birds that he could not tell which cockatoo he liked best. Here, however, in the District Nursing Association for which he was pleading was a cockatoo eminently worthy of their choice, and he hoped they would support it as it deserved. Even those who had been blessed in health must be able to appreciate the value of the ministrations of these nurses to the sick poor. The nurses had to become acquainted with night and storm as well as sunshine and daylight, and to face scenes from which brave men would recoil, but they had their reward—he made this remark because he saw many nurses before him—in the examples of self-sacrifice and heroism which they encountered in their daily work. This had a bracing and elevating effect on the character of the nurse, and led her and those with whom she came in contact to take a more optimistic view of life. The nurse, however, was, after all, very human in her needs; she required food, clothing, and housing, which must be provided by those who appreciated her services, whose gratitude was not like those autumn fruits which never ripened, but had a practical result.

Dr. Wells, district medical officer, testified to the great improvement in the dwellings of the poor effected by the nurses, and said that double the number was wanted.

Mr. Hayes Fisher, M.P., said that even the "nimble ninepence" need not be despised by collectors. He thought that everyone should take a share in this work, and that no one's education was complete unless they were helping to make this great city the fairest city on God's earth.

Mayor Skinner, for many years a member of the London School Board, spoke in warm terms of the nurses' work in the schools.

In reply to a vote of thanks, the chairman said that taking the income at £500, being the sum contributed by working men's clubs, concerts, etc., the cost of the nurses' visits was £1 for every 43 visits. This was very much below what should be paid. The two boroughs concerned must see that the Association was better equipped with funds to carry on its work for the sick poor.

### Everybody's Opinion.

[Correspondence on all subjects is invited, but we cannot in any way be responsible for the opinions expressed by our correspondents. No communication can be entertained if the name and address of the correspondent are not given as a guarantee of good faith, but not necessarily for publication. All correspondents should write on one side of the paper only.]

### DISTRICT NURSES AND PAYING PATIENTS.

Mrs. Llewellyn, Hon. Sec. of the Aberavon and Port Talbot District Nurses' Association, writes: This Association is not affiliated to the Queen's Jubilee Nurses.

### PROTECTING MATTRESSES.

**"G. H." writes: As matron of a small country hospital, I should be glad to learn through your pages the best way of protecting mattresses. Is it still generally considered sufficient to use the recognised mackintosh, sheet, and drawsheet, or is it usual to encase each mattress in a calico slip, removable for washing, like a pillow-slip.**

### THE MIDWIVES ACT.

"Gaute" writes:—It has struck me as very strange that a midwife can be registered and made a legalised practitioner without any reference to her private character whatever. All she has got to do is to send in her certificate—the L. O. S., may be—her signed identification paper, and 10s. 6d. In return for this she obtains a strip of parchment which practically authorises and encourages her to go on and prosper or the reverse without any supervision and armed with the protection which registration affords. Ought not some close investigation be made into the personal character of the applicant before she is recognised by the State and trusted by a helpless public as a registered midwife?

∴ The Midwives Act, 1902, provides that "any woman who . . . claims to be certified under this Act shall be so certified, provided she holds a certificate in midwifery from the Royal College of Physicians of Ireland, . . . or such other certificate as may be approved by the Central Midwives Board, or produces evidence, satisfactory to the Board, that at the passing of this Act she had been for at least one year in *bonâ-fide* practice as a midwife, and that she bears a good character."—Ed. of The Hospital.

### PORTRAITS AS PRESENTATIONS.

"A Sister" writes:—As one who has seen some very useless gifts made to matrons and other officials as marks of esteem, I rejoice to see the letter of C. H. advocating the presentation of miniature portraits. The editorial endorsement of the proposal should go a long way towards commending it not only to nurses, but also to persons in high quarters who have influence in such matters. I should like to say that, though I have no expectation of receiving a presentation myself, there would be nothing I should more thoroughly appreciate that the portrait of the matron under whom I have the privilege of serving.

### RESIGNATION OF NURSES AT AN IRISH INFIRMARY.

Nurses Murphy, Stuart, and Meagher write from 11, Homeville Place, Western Road, Cork, March 9th, 1904: With reference to a note which appeared in your paper a few weeks ago, relating to the resignation of three trained nurses in an Irish Workhouse Infirmary, in which it was stated that no explanation was given as to their actions. This we now proceed to give. In the first instance we entered under the heading of first-class rations, which were partly cut off, and inferior food supplied, which was considered quite good enough for us, who were doing twelve consecutive hours' night duty, with an average of one hundred and twenty patients each under our charge. Moreover, we were only allowed a bare fortnight's holiday in the year, and such a thing as getting off duty for even an hour was unheard of, unless we supplied nurses at our own expense, which was rather hard, considering that two of us were receiving a salary of £25 per annum each. When one of us, after two and a half years, applied for an increase of salary, it was not noticed; whereas when another person who was received into the infirmary with no experience in nursing whatever, and drawing the same salary after the same period of service, applied for an increase, it was immediately granted. After our resignations, the advertisement appeared for "qualified" nurses, offering a salary of £25 per annum, with £2 10s. increase yearly, rising to £35, which would not be granted to us trained nurses. Altogether, we considered ourselves badly treated, but of course our friends were not amongst the Board of Guardians.

### ASYLUM NURSES.

"A Matron" writes:—Relative to one of the "Notes on News from the Nursing World," in a recent issue of your journal concerning an asylum and asylum nurses, I, as a matron for many years in such an institution, should like to indicate the fallacy of some of the remarks made in the above. The writer of the Note points out, unknowingly or otherwise, that asylum nurses belong to the uneducated class. Not so! Numerous examination papers preparatory to their final, will distinctly prove otherwise. Also, as regards their general character and conduct, asylum nurses, from the nature of the cases that they have to deal with, have at times their patience tested to the utmost. Great tact, firmness, and kindness have always to be exercised. I must say that invariably I find in them all that could be desired where the interest of their patients is concerned. As regards the discipline, they are no more difficult to control than any other large body of nurses, and as to their training, they are given ample opportunities to claim for themselves, not only in ordinary nursing, afforded them in the infirmaries, but in the nursing of mental disease met with in the large blocks, an equality with any other branch of nursing. Therefore I maintain that asylum nurses are not in any way inferior to hospital nurses, but in many respects are as well educated, and certainly in some, better disciplined. I think that all should be done to encourage a body of women who, until recent years, have been so unnoticed. Discouragement is disheartening, and surely their work merits every consideration.

### Novelties for Nurses.

By Our Shopping Correspondent.

HYGIENIC CORSETS AT MESSRS. DEBENHAM'S.

Nurses engaged in private work will be glad to know of the new corsets recently invented by Messrs. Debenham and Freebody, as they are specially adapted for invalids and those requiring support. They are made of a peculiarly strong silky looking thread, which, while not elastic, gives to the figure. The bones in the back are strong, and thus yield support where it is most required. The corsets are slightly boned, so as to avoid undue pressure, and being designed with the straight-fronted bust, do not compress the waist. The patterns shown me by the manageress and corsetiere, Mlle. Zilva, were of charming blue and soft pink, woven in a fine mesh which had the appearance of knitting. One of these was a corset and hip-protector combined, and can be had either in one or in two parts, the upper consisting of a corselet and the lower covering the hips only. The cost of the combined corset, I was informed, is three guineas, the corselet being thirty-five shillings and the hip-protector thirty-eight shillings. The latter would be found useful as a support in exercises, riding, etc. The invalid's corset shown in the illustration, in blue, woven in strong silk, is made in three shapes, all straight-fronted, from 35s., and has suspenders attached. An abdominal belt is manufactured in the same material, and gives strong support, while it is at the same time soft and comfortable to the figure. These corsets are made to special medical order, from two guineas, and I am sure the manageress would take pains to carry out the required details, and to study individual needs. I was also shown some strongly woven shoulder straps, designed to prevent the unpleasant habit of stooping; and other productions to be obtained in this department are infants' belts. These are knitted, and the cost is 5s. 6d. Mlle. Zilva will be pleased to receive nurses requiring any of these articles, in the French corset department at Wigmore Street; and to show them her special designs.

### Appointments.

[No charge is made for announcements under this head, and we are always glad to receive, and publish, appointments. The information to insure accuracy should be sent from the nurses themselves, and we cannot undertake to correct official announcements which may happen to be inaccurate. It is essential that in all cases the school of training should be given.]

Fever Hospital, Hawick.—Miss Katherine Armitstead has been appointed matron. She was trained at the City Hospital, Edinburgh, and also at the Ladywell Hospital. For more than eight years she has been assistant matron at the City Hospital, Edinburgh.

Isolation Hospital, Crewe.—Miss E. B. Norris, who has held the post of matron of the Isolation Hospital, Crewe, since it was opened some six years ago, has been presented, on her resignation, with a marble clock and a purse of gold by the Corporation in recognition of her services. The hospital staff also presented her with a handsome silver coffee service as a mark of their regard.

Isolation Hospital, Menston, Yorks.—Miss Mary Munro has been appointed matron. She was trained at the London Hospital, London, where she was also staff nurse. Subsequently she was head nurse at the Goole Cottage Hospital, charge nurse at the Brook Fever Hospital, Shooter's Hill, London, matron at the Convalescent Home, Bona, Inverness, and sister at the Sanatorium, Chingford, Essex.

Nottingham Private Nursing Association.—Miss Jean Duncan has been appointed lady superintendent. She was trained at the Birmingham General Hospital. She has since done private nursing for three years; been nurse at St. Thomas's Hospital, London, charge nurse at St. George's Hospital, London, and matron of Dr. Jessop's Surgical Home at Leeds for eight years.

Stapleton Hospital, Fishponds, Bristol.—Miss Mary Light and Miss Edith A. Speary have been appointed assistant nurses. They were both trained at Stapleton Hospital.

Trowbridge and Melksham Union Infirmary.—Miss Florence Kite has been appointed superintendent nurse. She was trained at Greenwich Union Infirmary, and has since been ward nurse at Camberwell Union Infirmary, assistant nurse at Eastry Infirmary, superintendent nurse at Canterbury Infirmary, superintendent nurse at Ladywell Workhouse, and nurse at the Licensed Victuallers' School, Kennington Lane, London.

### Presentations.

City Hospital, Edinburgh.—Miss Katherine Armitstead, who is resigning her appointment as assistant matron of the City Hospital, Edinburgh, in order to take up the duties of matron of Hawick Fever Hospital, has been presented with a fitted dressing-case, a cream-jug, and a toast-rack as a token of affection from the nursing staff.

### A Book and its Story.

### NEW NOVEL BY MRS. BAILLIE REYNOLDS.*

Mrs. Baillie Reynolds' new novel is a well-balanced study of two temperaments: that of Phœbe Carburton, the heroine, and Donald Gilmour, whom she marries, but does not love. They met first at a dance, given at a house in the suburbs occupied by the parents of a member of the firm in which Gilmour was a junior partner, and by that encounter the whole tenor of Phœbe's life was changed. Gilmour had had but one love episode in his life, and since that, although he was now five-and-thirty, he had not again troubled himself about women until Phœbe crossed his path. Gilmour found that he and Phœbe's father had been at the same public school. Mr. Carburton was ten years his senior, and was already known as the first man of his year at Oxford when Gilmour was still a sixth-form boy. He remembered his name with pride as being one that had brought honour to the school, and for the moment, in looking at the girl's face, the scene of long ago came before his eyes again. "The headmaster entering wreathed in smiles; behind him a tall youth, beautiful as a Greek athlete, with the joy of life beaming in his eyes, the light of triumph in his face. He remembered even some of Carburton's words as he told his ambitions to a ring of sixth-form boys in the playground afterwards—visions of a rosy future sparkling in eyes that had been so like Phœbe's. This girl who sat beside him, a woman grown, was actually the daughter of a man whom he had almost considered a contemporary, between whose age and his own there was probably not more than ten years." Phœbe replied frankly to the many questions which Gilmour put to her, and he learned without difficulty that ill-health and an early marriage without means on either side had brought Bertram Carburton into a sphere very remote from that to which by birth and brains he was entitled and that he was living in the vicinity. It was a region remote from Gilmour's own town quarters, but the sudden interest which Phœbe and her belongings had aroused made him oblivious of everything but the wish to see her father, and, if possible, to be of use to him in his altered circumstances. "There was something gentle and unapproachable about Gilmour—a sweetness of manner which prevented the ordinary person from finding out what a reserved man he really was. Phœbe looked with approval at his kind blue eyes, and said she was sure her father would be pleased; but he imagined there was a shade or two of cordiality lacking in her voice, and wondered why. Naturally life on such a humble scale that even an unexpected visitor is disturbing and almost unwelcome did not present itself to him." Phœbe had told him that she had acted as an amanuensis, and had learned typewriting to assist her father in his literary work until his health failed. Now she was working for Mr. Gregson, their host, and her spare time was occupied in journalism. "I do things out of office hours," she said; "little essays about anything that strikes me, you know. Of course, father is a great help. I daresay it is a great deal the fact of having such a clever father that makes life seem so interesting." Gilmour made his call upon her parents, and won the children's hearts at once. He arranged a Hippodrome party for them on the following Saturday afternoon, when they and Phœbe were introduced to Lady Emma Knevett, whose husband was Gilmour's partner. She at once took them under her kindly wing, and Phœbe was asked to dine at "the comfortable, unpretentious house, in the unfashionable locality of Westbourne Terrace, where the Knevetts lived when in town. Joseph Knevett had married Lady Emma Gore, who was not only well born, but, what is somewhat rarer, well bred.

Phœbe, looking back, always thought of this dinner-party as a wonderful event in her life. She came into a circle which was by nature her own, and if she felt a little bewildered at first, "there was no consciousness at all in her smile, but a pleasure in seeing a room full of smart strangers. Lady Emma's friends were as a rule undeniable; but they were also, as a rule, smart." As a stranger in a set where everyone knew everyone else, Phœbe attracted some attention. To Gilmour she appeared, as she had done on the night of their first meeting, in totally different surroundings, distinctive with the distinction which a refined personality, and strong intellectuality united to a certain spirituality, give to its possessor. "The rose-coloured sunset was burning through the crisped edges of her hair, and the mass of coloured blossom just behind her shoulder in a large Oriental bowl seemed the very thing to accentuate and set off her pallor. He felt a curious wholly unexpected leap of heart as he saw her stand there, and thought her for a moment almost beautiful." Among other kind devices, Gilmour had invited Phœbe and her father to stay with him in the spring at a country cottage on the river. The Knevetts had a place close by, and Phœbe was by this time firmly established in Lady Emma's affection. Eric Waterson, the man who lived with him in town, "whose intense self-confidence and energy were in strong contrast to his friend, and whose views differed on almost every subject, and who yet, in some curious way, was necessary to him," was of the party. Waterson's character is one of the most interesting in the book. It is a well-drawn and consistently developed delineation. Phœbe throughout the book, in spite of some offers and many admirers, does not lose her heart to anyone. But after her father's death, which came suddenly, very soon after their visit to the cottage, Gilmour renewed the offer which he made to her then, and met with a refusal. Three months later he made a second attempt, and we read, "The offer was renewed and the girl unhesitatingly accepted it. This was what her father would have wished; this was as unlike all her own wishes as anything she could have devised." After a month's engagement Phœbe and Gilmour were married. "Up the church came the bride on Mr. Knevett's arm, preceded by eight small choristers, and followed by Winnie and Marjorie in their white and pink. There, at the chancel steps, stood the bridegroom and the best man close together, both with their eyes fixed upon the coming bride. Even in the temporary suspension of her mental faculties the girl said that the moment for retreat had gone by. She should never have arrayed herself and come to church if she really intended to throw up the part. It seemed to have resolved itself now into the question of whom she should betray—her mother and the children, who all relied upon her, or this man who was to be her husband." "It is no betrayal," she told herself. He knows I do not love him—he knows; he said it did not matter." From this point Mrs. Reynolds' clever story grows in force, and the real "study" in temperament begins.

* "Phœbe in Fetters." By Mrs. Baillie Reynolds. (John Murray. 6s.)

### Echoes from the Outside World.

### Movements of Royalty.

ON Thursday last week the forty-first anniversary of the wedding of the King and Queen was celebrated. An exceptionally large number of callers at Buckingham Palace signed their names in the visitors' book, and numerous congratulatory messages were received from various quarters. The Queen marked the occasion by presenting to his Majesty a very fine portrait of herself. The picture, which is in oils, depicts her Majesty in her coronation robes. Their Majesties practically spent the whole day at home, but dined with the Prince and Princess of Wales at Marlborough House in the evening.

On Tuesday the King inspected the model for the memorial to Queen Victoria at Mr. Brook's studio.

On Friday evening the Queen, accompanied by the Princess Victoria, and attended by Miss Knollys, went to St. Anne's, Soho, and was present at the performance of Bach's Passion music. A shortened form of the evening service was used. On this occasion the organ was augmented by an orchestra of 16 stringed and wind instruments, whilst the choir was proportionately strengthened. Mr. E. H. Thorne directed the choir, and the rendering of the devotional music was very impressive.

On Tuesday the Queen was present at a concert at Stafford House on behalf of Lady Henry Somerset's Industrial Farm at Duxhurst, Reigate.

### The Prince and Princess of Wales at Portsmouth.

FOLLOWING the King's visit, the Prince and Princess of Wales have paid a visit to Portsmouth, as the guests of Sir John and Lady Fisher. On Saturday afternoon they inspected the submarines and the *Victory*, subsequently proceeding to the Museum, where the Prince himself pointed out to the Princess the most notable objects, in which she was greatly interested. A display of signalling was given on the upper deck of the *Victory*, the special feature being the representation by a squad of signal boys of the exercises by which a fleet is manœuvred at sea. There was a dinner party at Admiralty House in the evening, and on Sunday morning the Prince and Princess attended service in the dockyard chapel. In the afternoon they made a tour of the dockyard in a train. On Monday the Prince of Wales witnessed a series of manœuvres from the *Mercury*. As his Royal Highness stepped on board his standard was broken at the main, and the Marine guards on board the *Victory* and *Hercules* saluted, these being the only signs of ceremony observed, and the *Mercury* at once slipped from the wharf and proceeded out of the harbour. The Princess of Wales went in the afternoon for a drive to the historic ruin of Porchester Castle. On Tuesday morning their Royal Highnesses were driven in an open carriage to the engineering depot of the Osborne Naval College. They also visited other parts of the College in Osborne grounds, as well as Osborne House.

On Wednesday the Princess of Wales laid the foundation stone of a new church at Eastney Barracks.

### Presentation to Miss Knollys.

AN interesting presentation was made last Friday night at Buckingham Palace by Mr. Walter M. Hitchcock, on behalf of the Geelong Fire Brigade, one of the best in Australia, who requested the acceptance by the Hon. Charlotte Knollys of a bronze medal framed in silver. The medal bears an inscription in appreciation of the presence of mind displayed by Miss Knollys on the occasion of the fire at Sandringham in December, and an expression of thankfulness for her timely warning to the Queen when she was in imminent peril.

### The Bombardment of Port Arthur.

A ST. PETERSBURG telegram gives official details of the latest bombardment of Port Arthur. The new town, it is stated, suffered the greatest damage. A shell burst eight yards from the house of a lawyer, and the wife of Colonel Baron Frank, who was in the house at the time, was struck by a number of fragments of the shell; and her daughter's head was blown off. The lawyer was killed on the spot, and another young lady was so badly injured in the right breast that she succumbed in the hospital to which she had been removed. The bombardment is described as "terribly severe."

### The New Governor of Bermuda.

THE King has approved of the appointment of Major-General Sir R. MacGregor Stewart, R.A., K.C.B., to be Governor and Commander-in-Chief of Bermuda, on the retirement of Lieut.-General Sir Henry Le Guay Geary, R.A., K.C.B. The new Governor was born in 1841, and entered the Royal Artillery in 1860. He has seen service in various parts of the world, including Afghanistan and Egypt, and has three times been mentioned in despatches. Sir Robert was from 1887 to 1897 aide-de-camp to her late Majesty Queen Victoria, and he has recently been commanding the Royal Artillery in the Southern Division.

### Miss Viola Tree's Début.

BOTH Mr. and Mrs. Tree have such a large circle of admirers amongst the theatre-going public that naturally much interest attached to the début of their daughter at Edinburgh last week. She appeared as "Viola" in "Twelfth Night," and came through the ordeal very successfully. Miss Tree has many of the gifts requisite for the profession upon which she has entered. She is tall, has a sweet face, and her voice is pleasant and well modulated. She evidently possesses much of the family talent, and sang an air, "Come Away, Death," creditably. She has also already mastered the art of walking gracefully on the stage, and betrayed no undue nervousness upon the trying occasion. Mr. Tree, with his daughter, came forward after the fall of the curtain, amidst a scene of enthusiasm, and the débutante was loaded with baskets and bouquets of flowers. In thanking the audience for the reception, Mr. Tree said that the splendid encouragement given to his child that night made him "very proud, and ought to make her very proud—and modest too."

### The Elgar Festival.

ON Monday evening the Opera House at Covent Garden was full from floor to ceiling for the first performance of the Elgar Festival, the audience including the King and Queen, the Princess Victoria, and Prince and Princess Charles of Denmark. The arrangement of the theatre was the same as that adopted for the fancy dress balls, and the difficulties of converting a theatre into a concert room acoustically satisfactory, are, of course, very great. On this occasion the choristers were so far back on the stage that they were unable to give full effect to their voices, and the blue canvas over their heads was also detrimental to their voices. Nevertheless they did their parts well, and the rendering of the "Dream of Gerontius" was thoroughly enjoyable. Mr. John Coates as "Gerontius" was magnificent, and the impressive utterances of the "Angel of Agony," as given by Mr. Ffrangcon Davies, singularly good. Mme. Kirby Lunn sang the beautiful music of the "Guardian Angel" with much tenderness and dignity, and the playing of the Manchester Orchestra, conducted by Dr. Richter was almost faultless. Dr. Elgar, who appeared on the stage at the close, was enthusiastically applauded. "The Apostles" was performed on Tuesday, and the King and Queen were again present, and the success achieved was beyond doubt.

### Notes and Queries.

**FOR REGULATIONS SEE PAGE 277.**

*Hospital Training.*

(215) Will you be good enough to tell me if the Victoria Park Hospital for Phthisis is a recognised school for training nurses?—*L. T.*

The City of London Hospital for Diseases of the Chest, Victoria Park, is a special hospital, and as such cannot give general training, but the authorities issue a certificate for two years which, supplemented by one year's training from an approved general hospital, is sometimes recognised as equivalent to a three-year certificate.

Will you kindly tell me of any hospitals, children's preferred, where probationers of 18 are taken?—*M. F.*

Hospitals which receive such young probationers are few and far between. Apply at the Bradford Children's Hospital, Bradford, and at the Victoria Home for Invalid Children, Margate.

*Abroad.*

(216) I should be obliged if you will kindly inform me if the "Holland Institute" is still in existence; if it is not, will you give me the address of any other institutions, not at Nice, which send nurses to France or Italy for a few months.—*E. A. C.*; and in Switzerland.—*L. N. C.*

The Holland Institute is now the Nice Nursing Institute. There is no agency for sending nurses abroad, but the English Nurses' Home, Villa Albany, Biarritz, the English Nurses' Institute and Medical Home, Sunny Bank, Via Borgo Pescio, San Remo, and the Anglo-American Nursing Home, Rome, and Association of Trained Nurses and Masseuses, 7 Via Rondinelli, Florence, all employ English nurses. So does the Davos' Invalids' Home, Davos Dorg, Switzerland.

*Home.*

(217) Can you tell me of an institution where a mother and daughter, aged 11, could be received for open-air treatment? They are extremely needy through sickness.—*Pansy.*

The special hospitals and homes for consumption are the only institutions open to needy phthisical patients, and the open-air treatment is available in most of them. The Manchester Hospital for Consumption, Hardman Street, Deansgate, Manchester, and the Hospital for Consumption and Diseases of the Chest, Mount Pleasant, Liverpool, seem the most likely institutions to help the cases you mention. Successful applicants usually have to wait a very long time for admission.

*Naval Hospital.*

(218) Will you kindly tell me if any special training is required to get into a naval hospital? I have had full general and fever training already.—*No Name, M. O'D.* and *D. L.*

No. Apply for form of application to Queen Alexandra's Royal Naval Nursing Service, to the Director-General, Medical Department of the Navy, Admiralty, Craven House, Northumberland Avenue, W.C.

*South Africa.*

(219) Will you kindly give me any information about the South African Expansion Emigration Committee, 47 Victoria Street, S.W.?—*K. K.*

It has been organised by experienced philanthropists under the auspices of members of the Government.

*Sir William Bennett on Private Nursing.*

(220) Will you tell me, please, if the Address of Sir William Bennett on private nursing, which appeared in The Hospital, *Nursing Section*, is likely to be published in pamphlet form.—*O. L. W.*

It is just published, and may be obtained, price 6d., or post free 7d., from the Scientific Press, 28 & 29 Southampton Street, Strand, W.C.

### Important Nursing Textbooks.

"The Nursing Profession: How and where to Train." 2s. net; 2s. 4d. post free.

"A Handbook for Nurses." By Dr. J. K. Watson. 5s. net; 5s. 4d. post free.

"Practical Guide to Surgical Bandaging and Dressings." By Wm. Johnson Smith, F.R.C.S. 2s. post free.

"The Nurses' Dictionary of Medical Terms and Nursing Treatment." By Honnor Morten. 2s. post free.

"The Human Body: its Personal Hygiene and Practical Physiology." By B. P. Colton. 5s. post free.

"Art of Feeding the Invalid." (Popular Edition). 1s. 6d. post free.

"On Preparation for Operation in Private Houses. By Stanhope Bishop, F.R.C.S. 6d. post free.

### For Reading to the Sick.

"SEND OUT THY PERFECT LIGHT."

Rise, rise above the mountains,
With healing on Thy wings;
Break into the dark chambers
Where pain in secret stings.

Come, while the morning tarries
Our waiting eyes to bless;
Look through the lowly lattice,
Bright Sun of Righteousness!

Out of the gloom we call to Thee
Out of the helpless night
Sun of world, Sweet Saviour,
Send out Thy perfect light.

*C. F. Alexander.*

To know that Love alone was the beginning of nature and creature, that nothing but Love encompasses the whole universe of things, that the governing Hand that overrules all, the watchful Eye that sees through all, is nothing but omnipotent and omniscient Love, using an infinity of wisdom, to save every misguided creature from the miserable works of its own hands, and make happiness and glory the perpetual inheritance of all the creation, is a reflection that must be quite ravishing to every intelligent creature that is sensible of it.—*Wm. Law.*

It is one of God's merciful ways that good can be worked out of all evil; but He alone claims the prerogative of doing this. If we attempt to hold the tangled skein of evil, and to wind its dark and slippery threads round the circumstances of our own life in order that good may come, we work out swiftly but surely our own destruction. But even sin cannot frustrate God's love; and from the day in which sorrow and disappointment and vain endeavour blighted man's life, it has been God's delight to change the bitter waters of Marah into living streams of blessing. The world may blame our errors and misunderstand our motives, but all honest effort is accepted by our Master and shall be recognised by Him at the last great day.

O God, turn us unto the fear and love of Thee; be pleased that we may be included in Thy goodness; and them that have bowed their heads under Thine Hand, do thou raise up in good works, adorn them in virtue, And may we all be made worthy of Thy kingdom which is in the heavens, through the goodwill of God. Amen.

I cannot feel
That all is well, when darkening clouds conceal
The shining sun;
But then, I know
He lives and loves; and say, since it is so,
Thy will be done.

*S. G. Browning.*

## Figures and Tables

**Figure f1:**